# Arthrogenic muscle inhibition: A prevalent issue in knee arthroplasty

**DOI:** 10.1002/ksa.12804

**Published:** 2025-07-24

**Authors:** Alexandre Le Guen, Sébastien Parratte, Vincent Marot, Régis Pailhé, Hasnae Ben‐Roummane, Emilie Bérard, Etienne Cavaignac

**Affiliations:** ^1^ Department of Orthopaedic Surgery Hôpital Pierre Paul Riquet, CHU de Toulouse Toulouse France; ^2^ International Knee and Joint Centre Abu Dhabi UAE; ^3^ Hospital Nostra Senyora de Meritxell Les Escaldes Andorra; ^4^ Clinique Aguilera RAMSAY Santé Biarritz France; ^5^ Research Methodological Support Unit (USMR), Department of Clinical Epidemiology and Public Health Toulouse University Hospital (CHU) Toulouse France; ^6^ Department of Clinical Epidemiology and Public Health CERPOP, INSERM‐University of Toulouse III, Toulouse University Hospital (CHU) Toulouse France

**Keywords:** arthrogenic muscle inhibition, knee arthroplasty, knee surgery, rehabilitation

## Abstract

**Purpose:**

Flexion contracture is a multifactorial complication after knee osteoarthritis and knee arthroplasty. Among the causes, arthrogenic muscle inhibition (AMI) has never been studied. It is a failure to achieve proper quadriceps motor activation, which can lead to flexion contracture due to hamstring contracture. In this study, we hypothesised that AMI is present in patients with knee osteoarthritis and after knee arthroplasty. The aims were to assess: (1) the prevalence of preoperative AMI, for patients without preoperative AMI, (2) the incidence of post‐operative AMI at 2 weeks, (3) its associated factors at 2 weeks and (4) the incidence 90 days after surgery.

**Methods:**

An international, prospective study enroled 341 patients undergoing knee arthroplasty across three centres. 316 patients met the inclusion criteria: symptomatic knee requiring unicompartmental, total or revision arthroplasty. Twenty‐five patients undergoing simultaneous bilateral procedures were excluded. Among the included patients, 275 patients without preoperative AMI were analysed for post‐operative incidence and associated factors. AMI was assessed using the SANTI classification on the day of surgery, at 15 days, and at 3 months. One patient was lost to follow‐up at 3 months.

**Results:**

Preoperative AMI ≥  1 was observed in 13% (95% confidence interval [CI] = 9–17). At 2 weeks post‐operatively, AMI ≥  1 occurred in 36% (95% CI = 30–42), with 13% showing AMI ≥  2, characterised by quadriceps inhibition and flexion contracture. Female gender (odds ratio [OR] =  2.81; *p* < 0.002), early post‐operative flexion contracture attitude such as keeping the knee bent, placing a pillow under the knee, or folding the hospital bed (OR =  5.89; *p* < 0.001), and high pain scores (OR = 13.57; *p* < 0.001) were significantly associated with AMI ≥  1 at 2 weeks. At 3 months, AMI ≥ 1 occurred in 12.4% (95% CI = 8.7–16.9).

**Conclusion:**

AMI is a prevalent issue both pre‐ and post‐operatively. Its incidence underscores the relevance of this condition; it should be considered in the management of post‐operative flexion contracture in knee arthroplasty.

**Level of Evidence:**

Level III, observation cohort study.

**Abbreviations**
ACL: anterior cruciate ligament
AMI: arthrogenic muscle inhibition
TKA: total knee arthroplasty
UKA: unicompartimental knee arthroplasty
VMO: vastus medialis oblique

## INTRODUCTION

One of the objectives of total knee arthroplasty (TKA) is to correct flexion deformity [12, 13, 19, 32]. It is a stiffness, defined as the inability to extend the knee joint, either actively or passively [28, 32]. Surgical techniques to address pre‐operative flexion contractures include: adequate bone resection, ligament releases, removal of posterior osteophytes, and posterior capsular releases [1]. Among the multifactorial causes of flexion contracture before and after knee arthroplasty, no studies to date have documented the role of arthrogenic muscle inhibition (AMI); this was the first study to investigate this complication in this patient population.

**Figure 1:**
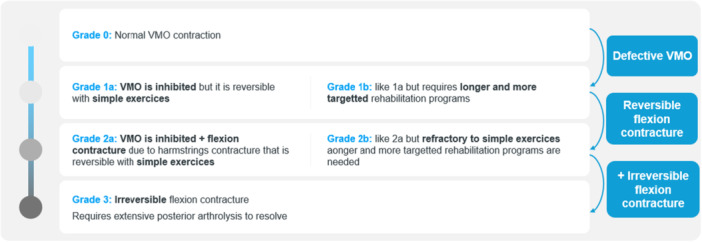
Clinical classification of arthrogenic muscle inhibition following knee injury or surgery. VMO, vastus medialis obliquus.

This phenomenon is a protective mechanism triggered by the injury to the periarticular muscles or joint structures that protects the muscles from increased stress after injuries [20, 25]. The activation of inhibitory interneurons reduces the efficiency of motor neuron pool recruitment, thereby decreasing muscle strength [16, 25]. The response of sensory receptors is altered, with profound effects on excitability in the primary motor cortex associated with a spinal reflex that typically produces a pattern of flexor facilitation and extensor inhibition [33, 35]. If AMI is not detected early, the hyperactivation of the hamstrings can lead to the risk of a fixed‐flexion contracture, with consequences such as altered gait and movement pattern, atrophy and chronic weakness of the quadriceps, dynamic instability, persistent knee pain, proprioceptive deficits and altered motor coordination [9, 25, 34]. In the context of the knee, AMI can be graded using the classification proposed by Sonnery Cottet et al. (Figure 1) [18, 33, 34].

The hypothesis of this study was that AMI can be observed in patients with knee osteoarthritis as well as after arthroplasty. As never described before on knee osteoarthritis and knee arthroplasties, we had several aims. First, to measure the prevalence of AMI preoperatively (AIM 1). Then, to measure the incidence of AMI at 15 days after knee arthroplasty in patients free of preoperative AMI (AIM 2 and main outcome) and to identify the associated factors for AMI 15 days after knee arthroplasty (AIM 3). Finally, we follow this cohort up to 3 months after surgery to measure the incidence of AMI (AIM 4).

## MATERIALS AND METHODS

From June 2023 to July 2024, a real‐world prospective international study was conducted in three centres in Toulouse, Abu Dhabi and Andorra. In this exploratory study, the goal was to include patients with a broad range of arthroplasty‐related conditions. Patients were included if they had symptomatic knee osteoarthritis requiring unicompartmental knee arthroplasty or TKA, or implant loosening requiring revision TKA. Patients who had undergone simultaneous bilateral arthroplasty were excluded.

**Figure 2:**
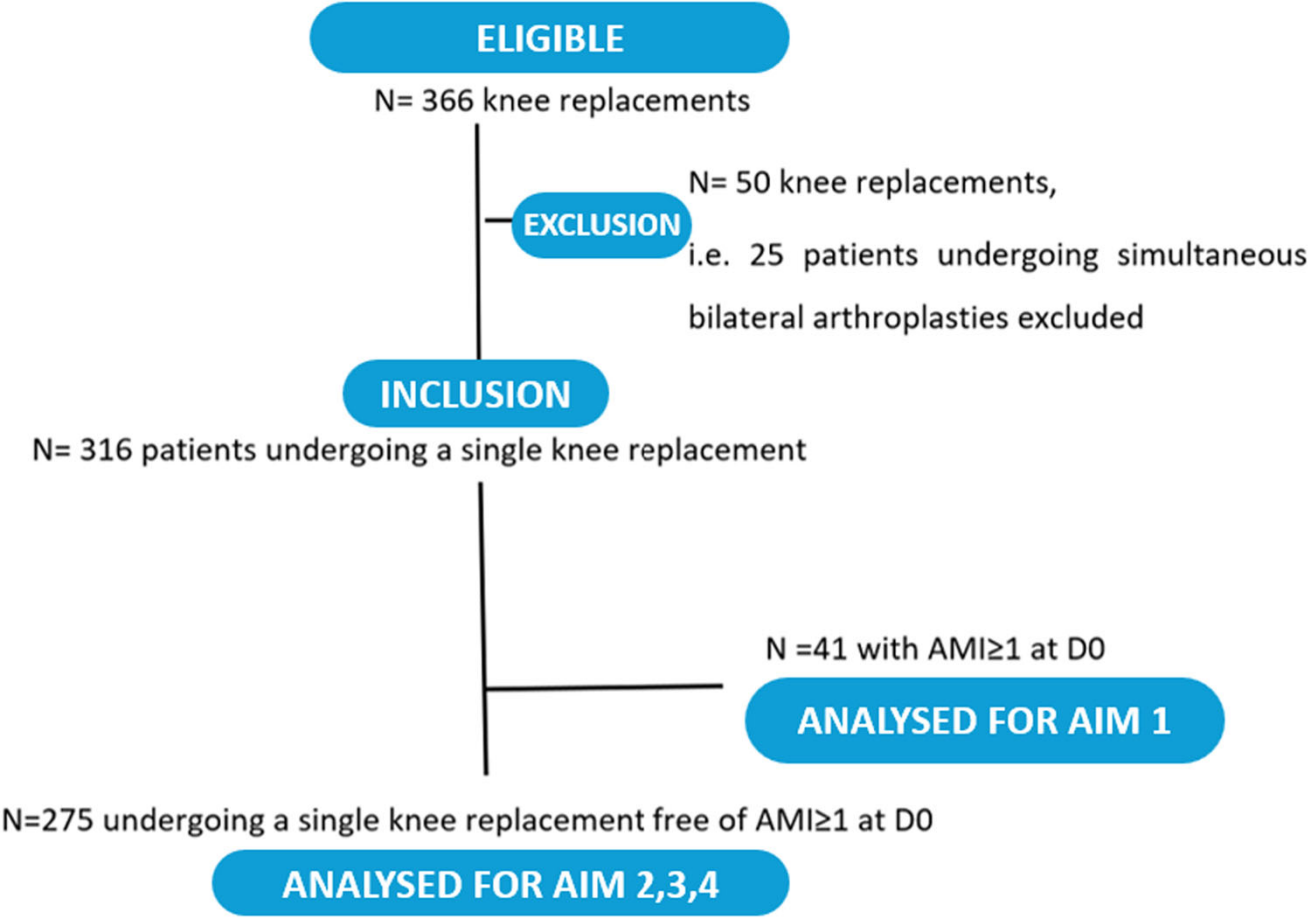
Flow diagram of patient selection for the analyses. AMI, arthrogenic muscle inhibition.

Of the 341 eligible patients, 25 had undergone simultaneous bilateral arthroplasty. Of the 316 patients who were included, 41 presented features of AMI preoperatively. Thus, post‐operative incidence of AMI and factors associated with post‐operative AMI ≥ 1 were analysed on 275 patients free of pre‐operative AMI (Figure 2).

**Figure 3:**
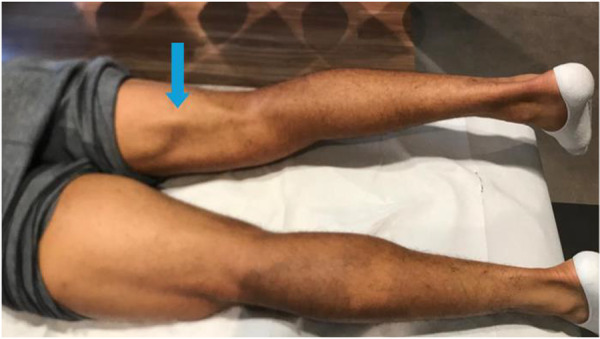
Fixed flexion contracture due to hamstring contracture, grade AMI 2. AMI, arthrogenic muscle inhibition.

Data were collected by three experienced knee surgeons at the three centres using the same standardised protocol. All patients included underwent a clinical examination by a knee surgeon and a knee X‐ray with anteroposterior view, lateral view and weight‐bearing flexed view of the knee. X‐rays were compatible with a diagnosis of symptomatic knee osteoarthritis or implant loosening for revision procedures. Clinical information such as: age, gender, body mass index, number of years of knee pain, number of physiotherapy sessions carried out pre‐operatively, preoperative knee injections, simple knee value score, knee surgical history (meniscectomy, anterior cruciate ligament [ACL] reconstruction, osteotomy, unicompartimental knee arthroplasty, tibial plateau fracture and TKA), and history of diabetes were collected [23]. Limb alignment (hip–knee–ankle angle) was measured on full‐length X‐rays based on a standardised protocol. The preoperative clinical examination included a standard knee examination with the range of motion with a goniometer, and the presence of laxity in full extension and thirty degrees of flexion and a specific assessment of AMI based on clinical features of vastus medialis obliquus (VMO) inhibition, presence of a hamstring contracture and reversibility of these features according to the clinical SANTI classification [33] (Figure 1). Although the SANTI classification was originally developed to assess AMI in patients with ACL injuries, it was employed in this study, as there is currently no validated or standardised tool available for evaluating AMI in the context of knee osteoarthritis or knee arthroplasty. The cause of flexion contracture is diverse in the arthroplasty population compared to the acute knee sprain population. In end‐stage knee osteoarthritis, flexion contracture is multifactorial, with bony osteophytes around the periphery of the joint, bony deformity, and posterior capsular tightness that would very commonly contribute to fixed flexion contracture [26, 32]. A flexion contracture was considered indicative of AMI when quadriceps inhibition was accompanied by a clinically visible and palpable hamstring contraction with the patient in the prone position (Figure 3).

Intra‐operatively, type of anaesthesia (spinal or general anaesthesia), approach (transquadricipital, midvastus or subvastus), and constraint of the prosthesis (Ultracongruent, Medial pivot, posterostabilised, hinge prosthesis, femoro tibial unicompartmental, femoropatellar unicompartmental or femoral reconstruction prosthesis) were recorded.

Post‐operatively, the patients were reviewed at 15 and 90 days after surgery. As a part of the systematic clinical follow‐up, the following factors were assessed: presence of AMI early flexion contracture attitude, presence of an effusion, and pain using the visual analogue scale (VAS) score [4].

As the incidence of AMI after knee arthroplasty has never been reported in the literature, we had to establish our hypothesis and calculate our sample size based on studies of AMI after ACL rupture [34]. Even if patients between arthroplasty and ACL rupture may be different, it was based on the best available data on AMI in the literature. The sample size was calculated with an expected incidence of 15 days post‐operative AMI of 50%, with a two‐sided 95% confidence interval (CI) with a width equal to ±6%, and needed 267 patients without preoperative AMI [22]. The CI is based on the exact (Clopper–Pearson) formula that uses the binomial probabilities directly. Additionally, patients presenting with AMI preoperatively and meeting eligibility criteria during the study period were also recruited for the assessment of the preoperative prevalence of AMI.

This multicentre prospective cohort study was approved by the local institutional review board in each of the three centres (RnIPH 2023‐26). Informed consent was obtained from all patients, and this study complied with the principles of the Helsinki Declaration.

Before analyses, verification of missing or aberrant or inconsistent data was conducted. After corrections, the database was locked. Analysis was performed on the locked database. We first described characteristics of patients using the appropriate descriptive statistics according to the type of variables. Descriptive statistics included mean with standard deviation (SD) for continuous variables and number of non‐missing observations with frequency (%) for categorical variables. Prevalence and incidence of AMI were calculated together with the corresponding exact 95% CI.

Categorical variables were compared between groups (with and without AMI ≥ 1 at 15 days after knee arthroplasty) using the chi‐square test (or Fisher's exact test when necessary). Student's *t* test was used to compare the distribution of continuous variables (or Mann–Whitney's test when distribution departed from normality or when homoscedasticity is rejected). The analysis of factors independently associated with AMI ≥ 1 at 15 days after knee arthroplasty was based on a logistic regression model including variables significantly associated with AMI in a univariate analysis (significance threshold of <0.20). The final model comprising the variables significantly (at the threshold of <0.05) and independently associated with AMI was obtained by a step‐by‐step, descending method. Intermediate nested models were compared using the likelihood ratio test. The goodness‐of‐fit of the model to the data was tested. All reported *p* values were two‐sided, and the significance threshold was <0.05. Statistical analyses were performed using STATA software 18.0 (STATA Corp.).

## RESULTS

**Figure 4:**
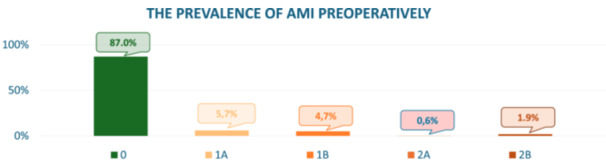
Spectrum and prevalence of different grades of AMI before a knee arthroplasty. AMI, arthrogenic muscle inhibition.

Overall, 13% (95% CI = 9–17) (*N* = 41/316) of the enroled population had AMI ≥ 1 preoperatively. The spectrum and prevalence of different grades of AMI, stratified according to the classification, are reported in Figure 4.

These patients were not included in the post‐operative analysis to clearly identify the post‐operative incidence of AMI ≥ 1 and associated factors. However, preoperative AMI ≥ 1 was significantly associated with 15‐ and 90‐day post‐operative AMI ≥ 1. Indeed, 75.6% of the patients with preoperative AMI ≥ 1 still had a 15‐day post‐operative AMI ≥ 1, while 36.0% of the patients with preoperative AMI < 1 developed a 15‐day post‐operative AMI ≥ 1 (*p* < 0.0001). Similarly, 32.5% of the patients with preoperative AMI ≥ 1 still had a 90‐day post‐operative AMI ≥ 1, while 12.4% of the patients with preoperative AMI < 1 developed a 90‐day post‐operative AMI ≥ 1 (*p* = 0.001). Moreover, among the six patients who were presenting a VMO deficit and a flexion contracture preoperatively, five of them were still presented a Stage 2 AMI at 15 days post‐operatively (4 Stage 2B, 1 Stage 2A and 1 Stage 0), and at 3 months, four were still presenting a Stage 2B, two resolved AMI.

**Figure 5:**
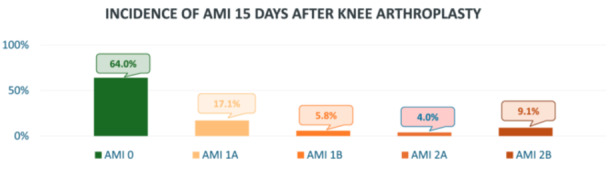
Spectrum and incidence of different grades of AMI 15 days after knee arthroplasty. AMI, arthrogenic muscle inhibition.

Among the 275 patients free of preoperative AMI, 36% (95% CI = 30–42) (*N* = 99/275) presented AMI ≥ 1, 15 days after surgery. Patient characteristics, stratified by the presence or absence of AMI ≥ 1 at this time point, are presented in Table 1, while the distribution of AMI grades at 15 days is shown in Figure 5.

**Table 1:** Study population, stratified by group (AMI or no AMI at 15 days post‐operatively).

	AMI ≥ 1 at D15			Total
	No	Yes		
	*N* = 176	*N* = 99	*p*	*N* = 275
Centre, *n* (%)			0.613	
Toulouse	138 (63.0)	81 (37.0)		219 (79.6)
Abu Dhabi	15 (62.5)	9 (37.5)		24 (8.7)
Andorra	23 (71.9)	9 (28.1)		32 (11.6)
Gender, *n* (%)			<0.0001	
Men	111 (72.5)	42 (27.5)		153 (55.6)
Women	65 (53.3)	57 (46.7)		122 (44.4)
Number of preoperative physiotherapy sessions (using quartiles) (q1–q2 vs. q3–q4), *n* (%)			0.103	
0–20	135 (66.8)	67 (33.2)		202 (73.5)
>20	41 (56.2)	32 (43.8)		73 (26.5)
Preoperative SKV score (using quartiles), *n* (%)			0.663	
0–20	54 (67.5)	26 (32.5)		80 (29.1)
25–30	38 (65.5)	20 (34.5)		58 (21.1)
35–50	53 (58.9)	37 (41.1)		90 (32.7)
60–100	31 (66.0)	16 (34.0)		47 (17.1)
Preoperative femorotibial deformity (HKA angle), *n* (%)			0.043	
>180	39 (54.2)	33 (45.8)		72 (26.2)
≤180	137 (67.5)	66 (32.5)		203 (73.8)
Preoperative flexion contracture, *n* (%)			0.422	
No	118 (62.4)	71 (37.6)		189 (68.7)
Yes	58 (67.4)	28 (32.6)		86 (31.3)
Duration of pre‐operative symptoms (using median, in years), *n* (%)			0.038	
1–6	108 (69.2)	48 (30.8)		156 (56.7)
>6	68 (57.1)	51 (42.9)		119 (43.3)
Preoperative injections, *n* (%)			0.683	
No	30 (66.7)	15 (33.3)		45 (16.4)
Yes	146 (63.5)	84 (36.5)		230 (83.6)
Knee history, *n* (%)			0.116	
No medical history	103 (62.8)	61 (37.2)		164 (59.6)
Meniscectomy	38 (66.7)	19 (33.3)		57 (20.7)
Uni or total arthroplasty	15 (88.2)	2 (11.8)		17 (6.2)
ACL reconstruction	13 (59.1)	9 (40.9)		22 (8.0)
Tibial plateau fracture	3 (33.3)	6 (66.7)		9 (3.3)
Osteotomy	4 (66.7)	2 (33.3)		6 (2.2)
Type of anaesthesia, *n* (%)			0.715	
General anaesthesia	100 (64.9)	54 (35.1)		154 (56.0)
Local anaesthesia	76 (62.8)	45 (37.2)		121 (44.0)
Approach, *n* (%)			0.335	
Subvastus	117 (66.9)	58 (33.1)		175 (63.6)
Transquad	50 (57.5)	37 (42.5)		87 (31.6)
Midvastus	9 (69.2)	4 (30.8)		13 (4.7)
Arthroplasty design, *n* (%)			0.485	
Ultracongruent	118 (61.1)	75 (38.9)		193 (70.2)
Medial pivot	13 (61.9)	8 (38.1)		21 (7.6)
Posterostabilised	12 (75.0)	4 (25.0)		16 (5.8)
Constrained	2 (100.0)	0 (0.0)		2 (0.7)
Hinge prosthesis	2 (100.0)	0 (0.0)		2 (0.7)
Femorotibial unicompartmental	26 (74.3)	9 (25.7)		35 (12.7)
Femoropatellar unicompartmental	3 (60.0)	2 (40.0)		5 (1.8)
Femoral reconstruction prosthesis	0 (0.0)	1 (100.0)		1 (0.4)
Arthroplasty design, *n* (%)			0.225	
Total knee arthroplasty	147 (62.6)	88 (37.4)		235 (85.5)
UniKnee arthroplasty	29 (72.5)	11 (27.5)		40 (14.5)
Diabetes, *n* (%)			0.927	
No	154 (63.9)	87 (36.1)		241 (87.6)
Yes	22 (64.7)	12 (35.3)		34 (12.4)
Post‐operative flexion contracture, *n* (%)			<0.0001	
No	143 (76.1)	45 (23.9)		188 (68.4)
Yes	33 (37.9)	54 (62.1)		87 (31.6)
Post‐operative effusion (using quartiles), *n* (%)			<0.0001	
0–1+	170 (66.9)	84 (33.1)		254 (92.4)
2–3+	6 (28.6)	15 (71.4)		21 (7.6)
VAS score at 15 days post‐surgery (using quartile), *n* (%)			<0.0001	
0–1	96 (95.0)	5 (5.0)		101 (36.7)
2	29 (74.4)	10 (25.6)		39 (14.2)
3–4	43 (56.6)	33 (43.4)		76 (27.6)
5–9	8 (13.6)	51 (86.4)		59 (21.5)

Abbreviations: ACL, anterior cruciate ligament; AMI, arthrogenic muscle inhibition; HKA, hip–knee–ankle; SKV, simple knee value; VAS, visual analogue scale.

At 15 days, results of the bivariate analysis demonstrated a significant association between AMI ≥ 1 and gender (*p* < 0.000), preoperative femoro‐tibial deformity (*p* < 0.043), duration of pre‐operative symptoms (*p* < 0.038), post‐operative flexion contracture (*p* < 0.0001) VAS score at 15 days after surgery (*p* <0.0001) and post‐operative effusion (*p* < 0.0001), as reported in Table 1.

**Figure 6:**
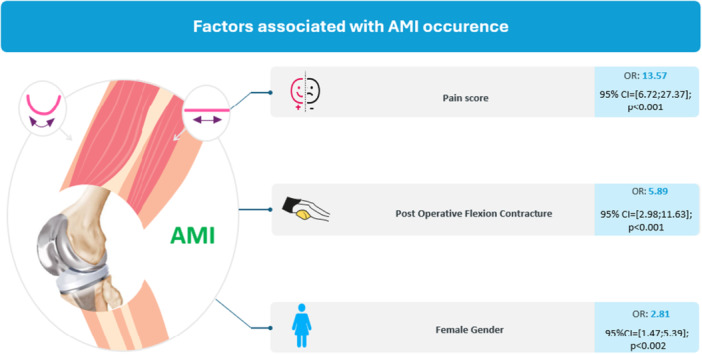
Factors independently and significantly associated with the occurrence of arthrogenic muscle inhibition (AMI ≥ 1) 15 days after a knee arthroplasty. CI, confidence interval; OR, odds ratio.

At 15 days after a knee arthroplasty, results of the multivariate analysis demonstrated that: a higher post operative pain score (OR = 13.57; 95% CI = [6.72–27.37]; *p* < 0.001), post operative flexion contracture (OR = 5.89; 95% CI = [2.98–11.63]; *p* < 0.001) and female gender (OR = 2.81; 95% CI = [1.47–5.39]; p < 0.002) were significantly and independently associated with incident AMI ≥ 1. Adjusted ORs in the multivariate model are summarised in Figure 6.

**Figure 7:**
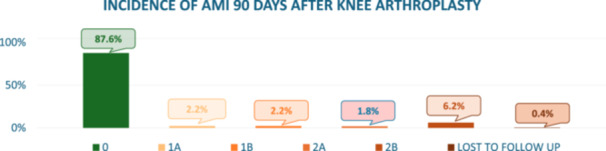
Spectrum and incidence of different grades of AMI 90 days after knee arthroplasty. AMI, arthrogenic muscle inhibition.

Among the 274 patients free of AMI ≥ 1 at D0 still followed up at 90 days, the incidence of AMI ≥ 1 was 12.4% (95% CI = 8.7–16.9) (*N* = 34/274). The spectrum and incidence of different grades of AMI are reported in Figure 7.

Of the 17 patients with Stage 2B after 3 months, 12 were already classified 2B 15 days after the surgery, 2 were classified 2A, 3 were classified 1B, 1 was classified 1A, and none of these patients were free of AMI at 2 weeks.

## DISCUSSION

The most significant outcome of this study was that AMI occurs not only following sports injuries, as previously documented in the literature, but also before and after knee arthroplasty [10, 17]. It was the first study to document a prevalence of 13% of AMI in knee osteoarthritis and an incidence of AMI of 36% at 2 weeks after knee arthroplasty, decreasing to 12.4% at three months. At 2 weeks, female gender (OR = 2.81; *p* < 0.002), early post‐operative flexion contracture attitude such as keeping the knee bent, placing a pillow under the knee or folding the hospital bed (OR = 5.89; *p* < 0.001), and high pain scores (OR = 13.57; *p* <  0.001) were significantly associated with AMI ≥ 1 at 2 weeks.

The prevalence of AMI following knee osteoarthritis (13%) cannot be compared to the literature because of a lack of data. Although the presence of a flexion contracture involving bone impingement, osteophytes, and several contractures affecting the posterior capsule, ligaments and tendons has been well described, no study has documented the flexion contracture following the lack of quadriceps activation [6, 24, 28].

The prevalence of AMI following end‐stage knee osteoarthritis appears to be considerably lower than that reported for flexion contracture, as AMI represents only a small component of the multifactorial aetiology of flexion contracture [32]. It is important to distinguish AMI from cases of flexion contracture where quadriceps activation remains satisfactory. Nevertheless, the observed prevalence can be compared in the literature to the much higher rate reported within six weeks following ACL rupture (56.7%) [34]. This rate difference is probably explained by the distinct pathophysiological mechanisms underlying AMI in chronic degenerative conditions versus acute ligament injuries [34]. In cases of ACL injury, shorter intervals between trauma and the initial outpatient evaluation have been identified as a risk factor for the development of AMI underscoring the critical role of the temporal dimension [34].

Without assessing the concept of AMI in arthroplasties, some studies were already interested in the influence of preoperative flexion contracture [12, 13]. This study found that most patients with preoperative flexion contracture due to AMI (Stage 2) still had it 2 weeks and 3 months after surgery. Given this, it seems logical to try to reduce the immediate and potential sequelae of AMI with evidence‐based intervention before surgery [8, 25].

The reported incidence of AMI ≥ 1, 15 days after knee replacement, was 36%. Some studies have focused on post‐operative quadriceps weakness due to the muscle's critical role in resuming functional activities like walking or climbing stairs, with strength loss nearing or exceeding 50% about 1 month after TKA [7, 15]. Nevertheless, quadriceps weakness has never been linked to hamstring fixed contraction after a knee arthroplasty. Studies have reported hamstrings strength deficit but not their pathological post‐operative contracture [7, 15, 21]. At Stage 2, hamstring contracture results in limited knee extension during gait, which Booij et al. showed to be highly significant with poor outcomes in knee replacement patients [5]. Without mentioning AMI, they explained limited knee extension as a result of impaired motor control with increased co‐contraction of the knee‐spanning flexor and extensor muscles [2, 5].

At 2 weeks post‐operatively, female gender emerged as a non‐modifiable risk factor associated with AMI following knee replacement. Concerning the modifiable factors, it seems essential to focus on the management of post‐operative pain. AMI is caused by changes in the discharge of articular sensory receptors (attributed to inflammation, pain and swelling), which in turn alter neurological pathways, resulting in a hamstring contracture and a quadriceps activation failure [3, 9]. This disruption in sensory feedback has repercussions on somatic and cortical dysfunction, with a reduction in the excitability of quadriceps motorneurons [9]. We can therefore understand the crucial role of pain management when we know that the prefrontal cortex is the area of the brain responsible for executive functions and pain processing [27]. It is relevant to note that this factor is similar to those described for stiffness after TKA, and effective analgesia can help prevent the occurrence of a stiff TKA [31]. Another treatment option would be to prevent temporary flexion contracture. After a cruciate ligament rupture, we found that patients who used a pillow under their knee or folded the hospital bed to relieve their pain had a higher risk of developing AMI [34]. Post‐operative analgesic flexion contracture must be addressed to prevent it from becoming permanent.

Finally, the incidence of AMI after knee arthroplasty decreased over time, from 36% at 15 days to 12% at 3 months. Among patients with features of AMI at 3 months, all of them were already presenting AMI 15 days after surgery. Most (62%) were at the same stage of AMI at both assessments. This finding underscores the importance of early detection of AMI, as these patients are at increased risk of developing AMI during follow‐up and, consequently, of experiencing stiffness‐related complications. These results point in the same direction as the studies which reported that the main factor predisposing to flexion contracture after TKA is preoperative flexion contracture [32].

The strength of this study is its multicentre prospective design. The number of patients studied and the low number of people lost to follow‐up can be highlighted. Moreover, multivariate analysis identifying factors significantly and independently associated with the 15‐day incidence of AMI, in AMI‐free patients prior to surgery, showed a good model fit (pseudo *R* ^2^ = 0.33). This multivariate analysis did not identify associated factors that were found to be significant in the bivariate analysis. The duration of preoperative symptoms, intra‐articular effusion, and preoperative intra‐articular deformity should therefore be investigated in future studies to determine whether they are truly associated with AMI ≥ 1 after a knee arthroplasty. Finally, given that AMI is a time‐sensitive complication, the study incorporated early post‐operative follow‐up with two scheduled assessments [34]. Nevertheless, as this is a novel concept in the context of knee arthroplasty, further long‐term studies are needed to determine whether the associated factors change in relevance over time and to explore the relationship between AMI and long‐term functional outcomes.

The main limitations of this study are related to the fact that AMI was only diagnosed based on the clinical assessment of arthrogenic inhibition of the vastus medialis obliquus using the classification of Sonnery Cottet et al. [33]. Some publications used surface EMG to objectively measure activation of the vastus medialis obliquus [11, 14, 17, 30]. EMG can be measured directly by collecting interpolated contractions or superimposed bursts or indirectly by measuring voluntary quadriceps strength using dynamometry [29]. One may wonder whether doing a visual evaluation only might make the diagnosis inaccurate. Nevertheless, surface EMG has inherent limitations and cannot accurately measure the activity of deep muscles [17]. The soft tissue between the electrode and target muscle can introduce interference, and the electrode could be mispositioned [17]. The measured muscle activity can also vary depending on the patient's position during the exam, and there are no EMG reference values (μV) [17, 30]. Conversely, the SANTI classification, without EMG assessment, has excellent agreement when evaluated blindly by various examiners [18]. The advantage of an exclusive clinical classification is that it is easy to use in daily practice by any healthcare professional, without devices or additional cost. Also, the examiner can detect substantial clinical improvements that could have been missed if we relied solely on EMG values. To date, this classification has only been applied in the context of ACL rupture and reconstruction. As the first study to investigate this phenomenon in patients with knee osteoarthritis undergoing arthroplasty, we used this classification as a pragmatic framework for initial clinical assessment, primarily due to the lack of a validated and standardised tool for diagnosing AMI in this population. Additionally, given the lack of data on AMI in arthroplasty patients, the sample size was estimated based on studies involving ACL injuries. This exploratory study provides a foundation for future research on AMI in patients undergoing knee arthroplasty.

Another limitation is the distribution of patient recruitment by centre. Most of the patients came from Toulouse. One of the reasons for this imbalance is the exclusion of 50 simultaneous bilateral arthroplasties, which significantly reduced the number of arthroplasties included in Abu Dhabi. As only performed in one out of three centres, simultaneous bilateral arthroplasties were excluded to avoid a centre effect. The patients included were therefore the target population, patients undergoing single knee arthroplasty, the most common case a knee surgeon has to deal with in daily practice.

Moreover, the incidence of Grade 3 AMI was not studied because its diagnosis requires considerably longer durations of follow‐up that were beyond the scope of this study. Finally, revision arthroplasties were included, even though they could be considered a distinct condition. In this exploratory study, the goal was to include patients with a broad range of arthroplasty‐related conditions, and this study provides a useful starting point for detecting and treating AMI in arthroplasty patients.

## CONCLUSION

This study showed that AMI occurs not only in patients who are candidates for knee arthroplasty but also post‐operatively. The relatively high incidence observed, with severe cases with persistent fixed flexion contracture, highlights the fact that this condition needs to be considered before and after knee arthroplasty.

## AUTHOR CONTRIBUTIONS

All authors contributed to the creation of this study. Designed the study, interpreted the data, wrote and reviewed the manuscript: Alexandre Le Guen. Designed the study and reviewed the manuscript: Sébastien Parratte, Vincent Marot and Régis Pailhé. Contributed to the data analysis and reviewed the manuscript: Hasnae Ben‐Roummane. Designed the study, contributed to the data analysis and reviewed the manuscript: Emilie Bérard. Designed the study, supervised the study, edited and reviewed the manuscript: Etienne Cavaignac. All authors have read the final manuscript and approved the manuscript for publication.

## CONFLICT OF INTEREST STATEMENT

Sébastien Parratte reports consultancy fees and royalties from Zimmer Biomet not related to this study. Régis Pailhé reports consultancy fees from Stryker not related to this study. Etienne Cavaignac reports consultancy fees and royalties from Arthrex and Amplitude, not related to this study. The remaining authors declare no conflicts of interest.

## ETHICS STATEMENT

This multicentre prospective cohort study was approved by the local institutional review board in each of the three centres. This research study complied with the principles of the Helsinki Declaration. Informed consent was obtained from all patients.

## Data Availability

Data are available upon reasonable request.
